# Invasive group A streptococcal infections requiring paediatric intensive care in children and adolescents: a nationwide retrospective cohort study in Austria

**DOI:** 10.1007/s00431-026-07280-z

**Published:** 2026-07-29

**Authors:** Anna Aichinger, Francesco Cardona, Judith Rittenschober-Böhm, Sophie Jansen, Sabine Kipp-Urlicic, Lorenz Stana-Hackenberg, Birgit Willinger, Angelika Berger

**Affiliations:** 1 Department of Pediatrics, Division of Neonatology, Pediatric Intensive Care and Neuropediatrics, Medical University of Vienna, Waehringer Guertel 18-20, 1090 Vienna, Austria; 2 Clinic for Neonatology, Kepler University Hospital Linz, Med Campus IV., Krankenhausstraße 26-30, 4020 Linz, Austria; 3 Department of Pediatrics and Adolescent Medicine, PICU, Klinik Donaustadt, Langobardenstraße 122, 1220 Vienna, Austria; 4 Clinic of Pediatrics and Adolescent Medicine, University Hospital of Salzburg, Müllner Hauptstraße 48, 5020 Salzburg, Austria; 5 Division of Clinical Microbiology, Medical University of Vienna, Waehringer Guertel 18-20, 1090 Vienna, Austria

**Keywords:** Invasive *group A streptococcal* infection, Paediatric intensive care, Septic shock, Respiratory infection, Extracorporeal support

## Abstract

Several European countries reported a marked rise in paediatric invasive *group A streptococcal* (iGAS) infections from mid-2022 onwards. We describe the clinical spectrum and intensive care course of children with iGAS admitted to Austrian paediatric intensive care units (PICUs) during this period. We conducted a nationwide, multicentre, retrospective observational cohort study across all 13 Austrian PICUs. Children aged 0–18 years admitted between 1 September 2022 and 31 March 2024 with microbiologically confirmed iGAS were identified using a standardised, pseudonymised case report form. Data on demographics, clinical presentation, organ support, co-infections, microbiology, and outcomes were collected. All 13 Austrian PICUs participated, contributing 70 cases. The most frequent initial symptoms were pleural empyema (51.4%), pneumonia (45.7%), sepsis (44.3%), and septic-shock (25.7%). Co-infections (viral/bacterial) were documented in 55.7%. Organ support requirements were substantial: 72.9% required mechanical ventilation (44.3% invasive; 28.6% non-invasive), 50.0% received vasoactive therapy, and 14.3% underwent cardiopulmonary resuscitation. Extracorporeal therapies were used in 10.0% overall, including extracorporeal membrane oxygenation and continuous renal replacement therapy. Microbiological confirmation most commonly derived from sterile sites, particularly pleural fluid and blood cultures. Overall mortality was 14.3%.

*  Conclusion*: During the 2022–2024 surge, iGAS presentations requiring PICU admission in Austria were predominantly respiratory, with high rates of organ support and notable mortality. These findings highlight the need for early recognition of respiratory-focused iGAS, careful assessment for co-infections, and timely access to advanced organ support in experienced centers. Prospective surveillance incorporating standardised diagnostics and treatment data is warranted.

**Table:** 

What is Known: • *Since mid-2022, several European countries have reported a resurgence of paediatric invasive group A streptococcal infections (iGAS) with increased disease severity. iGAS can lead to rapid clinical deterioration, frequently requiring intensive care and advanced organ support*.	
What is New: • *This nationwide multicentre study describes the full clinical spectrum and intensive care course of paediatric iGAS cases admitted to all Austrian PICUs during the 2022–2024 surge. Respiratory-focused disease predominated, with high rates of mechanical ventilation, vasoactive support, extracorporeal therapies, and a notable mortality.*.

What is Known:

• *Since mid-2022, several European countries have reported a resurgence of paediatric invasive group A streptococcal infections (iGAS) with increased disease severity. iGAS can lead to rapid clinical deterioration, frequently requiring intensive care and advanced organ support*.

What is New:

• *This nationwide multicentre study describes the full clinical spectrum and intensive care course of paediatric iGAS cases admitted to all Austrian PICUs during the 2022–2024 surge. Respiratory-focused disease predominated, with high rates of mechanical ventilation, vasoactive support, extracorporeal therapies, and a notable mortality.*.

## Introduction

*Group A Streptococcus (GAS*, *Streptococcus pyogenes*) is a Gram-positive bacterium well adapted to the human host, functioning as both a commensal and a pathogen. It primarily colonizes the pharynx, often in asymptomatic individuals [1–4]. GAS is responsible for a broad spectrum of clinical manifestations, ranging from asymptomatic carriage and post-streptococcal immune complications to severe, life-threatening invasive infections [5–8].

Invasive GAS infection (iGAS) is associated with substantial global mortality [2, 9, 10]. Reported case fatality in high-income settings typically ranges from 8 to 16% [6, 11]. Mortality can increase markedly when definitive antimicrobial therapy and toxin-modulating adjuncts (e.g. clindamycin and intravenous immunoglobulin) are delayed, particularly in case of streptococcal toxic shock syndrome (STSS) [11–14].

Following the onset of the COVID-19 pandemic, several European countries and the United States noted a decline in iGAS activity during 2020–2021 [15]. From mid-2022 onwards, as public health restrictions eased, a marked increase in paediatric iGAS with high admission rates and fatalities was reported, especially among children under 10 years of age [16–22]. Health authorities, including World Health Organization (WHO) and European agencies, issued advisories urging vigilance, rapid diagnosis and treatment, and increased public and professional awareness [15, 20, 21, 23–25].

Against this background, we sought to characterize the national burden of severe paediatric iGAS requiring paediatric intensive care in Austria. Our objective was to describe the clinical presentation, co-infections, microbiology, organ-support requirements, and short-term outcomes among children and adolescents admitted to Austrian PICUs between autumn/winter 2022 and spring 2024.

## Methods

### Study design and study group

This nationwide multicentre, retrospective, observational study included paediatric patients aged 0–18 years with a proven invasive *Group A Streptococcus* (iGAS) infection admitted to any of the 13 PICUs in Austria between 1 September 2022 and 31 March 2024. The study period was selected to capture the early post-COVID-19 iGAS surge and to include two autumn/winter periods in Austria, a time of increased transmission of respiratory pathogens.

Data were collected using a pseudonymised, standardised electronic questionnaire developed and administered via a university-licensed SociSurvey platform (SociSurvey GmbH, Munich, Germany) and distributed to all PICUs nationwide. Centres reported the total number of iGAS admissions and associated fatalities during the study period, along with detailed clinical, microbiological, and treatment data for each case. Initial clinical presentations and diagnoses were extracted from clinician-documented diagnoses in the medical records using the standardized questionnaire. Vasoactive support was recorded as documented treatment with vasoactive or inotropic medication during the PICU course. The cohort was restricted to patients admitted to the PICU; children who died before PICU admission were not captured.

### Diagnostic criteria for iGAS infection

Diagnostic criteria for iGAS infection were defined according to the Health Protection Surveillance Centre (HPSC), Ireland [26]. A case was considered confirmed when GAS was isolated from a normally sterile body site by culture or molecular methods (i.e. PCR), or when GAS was detected from a non-sterile site and the clinical criteria for a severe iGAS infection were fulfilled. Clinical criteria included a severe presentation consistent with iGAS or severe GAS infection, such as STSS, necrotising fasciitis, pneumonia, septic arthritis, meningitis, peritonitis, osteomyelitis, myositis, puerperal sepsis, or cellulitis, accompanied by a systemic condition requiring hospitalisation [26].

### Statistical analysis

Depending on the distribution, mean values and standard deviations or medians and interquartile ranges were calculated for numerical data. Categorical data were presented as absolute and relative frequencies. All statistical analyses were carried out using IBM SPSS Statistics and Microsoft Excel.

### Ethics

The study was approved by the Medical Ethics Committee of the Medical University of Vienna, Austria (EK Nr: 1273/2024).

## Results

### Study population

A total of 70 patients admitted across all 13 Austrian PICUs were included during the study period (Table 1). Slightly more than half were male (38/70, 54.3%).

**Table 1:** Baseline demographics and characteristics of children and adolescents with iGAS infection admitted to Austrian PICUs

	all patients
Total number of patients, n (%)	70 (100%)
sex, n (%)	
male	38 (54.3%)
age (years), median [IQR]	4.5 [2.0, 7.25]
year of admission, n (%)	
2022	9 (12.9%)
2023	52 (74.2%)
2024	9 (12.9%)
province, n (%) (proportion of the Austrian population)^ß^	
Vienna (22%)	32 (45.7%)
Salzburg (6%)	20 (28.6%)
Upper Austria (17%)	7 (10.0%)
Tyrol (8%)	4 (5.7%)
Carinthia (6%)	3 (4.3%)
Vorarlberg (4%)	2 (2.9%)
Lower Austria (19%)	1 (1.4%)
Styria (14%)	1 (1.4%)
Burgenland^$^ (3%)	
co-infection, n (%)	39 (55.7%)
pre-existing illness, n (%)*	8 (11.4%)
pulmonary	0 (0.0%)
cardiac	1 (1.4%)
former preterm baby	0 (0.0%)
metabolic	1 (1.4%)
neurologic	1 (1.4%)
other^+^	3 (4.3%)
combined^#^	2 (2.9%)

*iGAS* invasive group A streptococcus, *IQR* interquartile range, *PICU* paediatric intensive care unit

Values are n (%) unless stated otherwise; medians [IQR]

ß source: Statistics Austria www.statistik.at

$ there is no PICU in Burgenland; patients requiring PICU care are transferred to other Austrian federal states, predominantly Vienna, Lower Austria, or Styria.

*multiple answers were possible

+ patients with other pre-existing illness: trisomy 21; thrombocytopenia; Klippel-Trenaunay-Syndrome DD Kasabach Merritt Syndrome,

# combined pre-existing illness: former preterm baby + unknown syndrome (chylothorax, hypogammaglobinemia); neurologic + tuberous sclerosis and therapy-resistant epilepsy

Cases were reported from all Austrian federal states with participating PICUs, with the largest proportions from Vienna (32/70, 45.7%) and Salzburg (20/70, 28.6%).

With regard to pre-existing illness, multiple answers were possible. 8/70 patients (11.4%) had a documented pre-existing illness.

### Seasonality

**Fig. 1:**
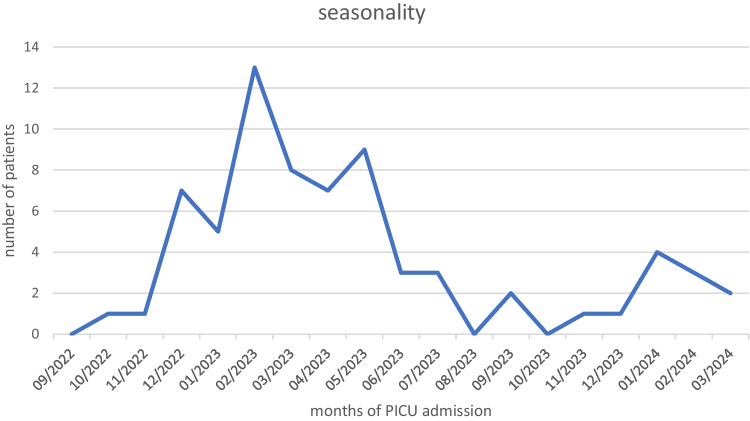
Monthly iGAS cases

Monthly case numbers increased from December 2022, reaching a peak in February 2023 (n = 13), followed by a gradual decline. From August 2023 onwards, only low monthly case numbers were observed**.** The observed peak therefore mainly reflected winter 2022/2023 rather than a consistent winter predominance across the entire study period **(**Fig. 1**).**

### Initial clinical symptoms at presentation

**Fig. 2:**
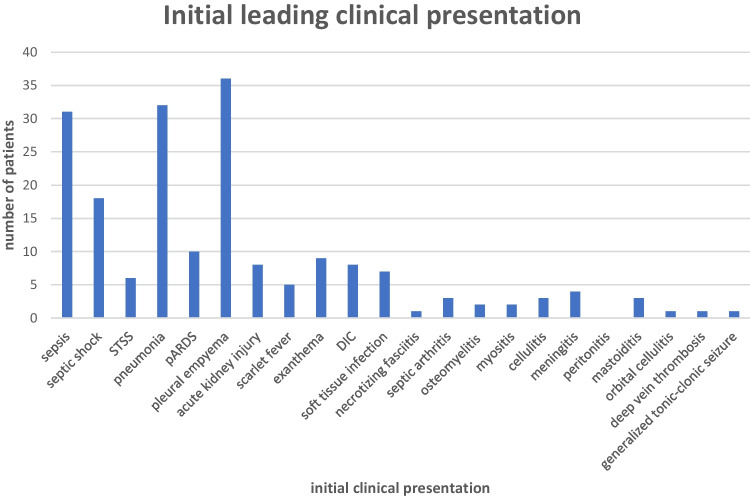
Initial clinical symptoms at presentation in children and adolescents with iGAS (n = 70). Multiple symptoms per patient were permitted. STSS = streptococcal toxic shock syndrome; pARDS = paediatric acute respiratory distress syndrome; DIC = disseminated intravascular coagulation

Multiple presenting symptoms were recorded per patient; 56/70 (80.0%) had ≥ 2 symptoms at presentation. The most frequent were pleural empyema (36/70, 51.4%), pneumonia (32/70, 45.7%), and sepsis (31/70, 44.3%). Other common presentations included septic shock (18/70, 31.0%), streptococcal toxic shock syndrome (STSS; 6/70, 10.3%), soft-tissue infections (7/70, 12.1%), paediatric acute respiratory distress syndrome (pARDS, 10/70, 17.2%), acute kidney injury (8/70, 13.8%), and disseminated intravascular coagulation (DIC, 8/70, 13.8%) **(**Fig. 2**)**. Figure 2 displays the number of patients in bar charts. Multiple responses per patient were possible.

### Critical care support

Most patients received mechanical ventilation (51/70, 72.9%); 31/70 (44.3%) underwent invasive ventilation and 20/70 (28.6%) received non-invasive ventilation. Half required vasoactive support (35/70, 50.0%). Extracorporeal therapies were used in 7/70 (10.0%) overall: continuous renal replacement therapy (CRRT) in 2/70 (2.9%), veno-arterial ECMO (VA-ECMO) in 2/70 (2.9%), and combined VA-ECMO + CRRT in 3/70 (4.3%). Cardiopulmonary resuscitation was performed at some point during hospital stay in 10/70 (14.3%).

PICU length of stay was a median of 12.5 days [IQR 6.0, 18.0], and 10 patients died (14.3%): seven within 24 h, two between days 2–7, and one after nine months of intensive care following *GAS* meningitis **(**Table 2**).**

**Table 2:** Critical care support and outcomes among children and adolescents with iGAS admitted to Austrian PICUs

	all patients (n = 70)
duration of PICU stay (days), median [IQR]	12.5 [6.0, 18.0]
mortality, n (%)	10 (14.3%)
mechanical ventilation, n (%)	51 (72.9%)
non-invasive ventilation	20 (28.6%)
invasive ventilation	31 (44.3%)
vasoactive support, n (%)	35 (50.0%)
extracorporeal support, n (%)	7 (10.0%)
CRRT	2 (2.9%)
VA-ECMO	2 (2.9%)
VV-ECMO	0 (0.0%)
Apheresis	0 (0.0%)
combination (CRRT + VA ECMO)	3 (4.3%)
CPR, n (%)	10 (14.3%)

*CRRT* continuous renal replacement therapy, *VA-ECMO* veno-arterial ECMO, *VV-ECMO* veno-venous ECMO, *CPR* cardiopulmonary resuscitation, *IQR* interquartile range, *PICU* paediatric intensive care unit

Values are n (%) unless stated otherwise; medians (IQR)

### Microbiology results

Microbiological confirmation was available for all patients (Table 3). Positive specimens were identified from both sterile and non-sterile sites, and patients could contribute more than one site. Among sterile sites and single positive samples, pleural fluid was the most frequent source (32/70, 45.7%), followed by blood culture (22/70, 31.4%) and cerebrospinal fluid (2/70, 2.9%); no positive results were obtained from ascites. Among non-sterile sites, the highest numbers of positive specimens were from throat swabs (13/70, 18.6%), tracheal secretions (11/70, 15.7%), wound swabs (8/70, 11.4%), and bronchial secretions (6/70, 8.6%).

**Table 3:** Documented positive microbiological results among children and adolescents with iGAS

	all patients (*n* = 70)
Microbiology, *n* (%)	
single positive samples	42 (60.0%)
combination (multiple positive samples)	28 (40.0%)
Sterile sites, *n* (%)	
blood culture	22 (31.4%)
pleural fluid^+^	32 (45.7%)
ascites fluid^+^	0 (0.0%)
cerebrospinal fluid^+^	2 (2.9%)
Non-sterile sites, *n* (%)	
urine culture	0 (0.0%)
tracheal secretions^+^	11 (15.7%)
bronchial secretions^+^	6 (8.6%)
wound swab^+^	8 (11.4%)
throat swab^+^	13 (18.6%)
nose swab^+^	1 (1.4%)
ear swab^+^	1 (1.4%)
ear and brain swab^+^	1 (1.4%)
nose and rectum swab^+^	1 (1.4%)
rapid test for *Group A* *streptococcus*	14 (20.0%)

Multiple positive specimens per patient were possible. Sterile sites include blood, pleural fluid, cerebrospinal fluid, and ascites

Only documented positive microbiological test results were recorded; negative results and total tests performed were unavailable. Therefore, percentages in this table refer to the proportion of patients with documented positive results within the cohort and should not be interpreted as test-positivity rates

Values are n (%) unless stated otherwise

+ Results reflect culture and/or PCR positivity

Testing strategies (and timing) were not standardized across centres

Multi-site positivity was observed in 28/70 (40%). The distribution of positive sites per patient was as follows: one patient had five positive sites, one had four, nine had three, and 17 had two. Among patients with multi-site positivity (n = 28), the most frequent contributing sites were pleural fluid (16/28, 57.1%) and blood culture (14/28, 50.0%); throat swab (9/28, 32.1%) and rapid group A streptococcal test (9/28, 32.1%) were also frequent contributors **(**Table 3**).**

### Co-infections (Pathogen Spectrum)

Co-infection (viral and/or bacterial) was present in 39/70 (55.7%); Viral co-pathogens included varicella-zoster virus (7/70, 10.0%), influenza A (8/70, 11.4%), human metapneumovirus (7/70, 10.0%), rhino-/enterovirus (5/70, 7.1%), influenza B (4/70, 5.7%), respiratory syncytial virus (2/70, 2.9%), coronavirus (1/70, 1.4%), herpes simplex virus 1 (1/70, 1.4%), and human parainfluenza virus (1/70, 1.4%). Bacterial co-pathogens occurred in 7/70 patients (10.0%) and included *Streptococcus pneumoniae* (2 cases), *Moraxella catarrhalis* (2 cases), and single cases of *Staphylococcus aureus/hominis*, *Streptococcus agalactiae* and *Haemophilus influenzae*. Mixed viral or viral-bacterial co-infection was identified in 4/70 (5.7%). Further details are provided in Table 4.

**Table 4:** Co-infections among children and adolescents with iGAS admitted to Austrian PICUs

	all patients (*n* = 70)
co-infection, n (%)	39 (55.7%)
viral co-infections, n (%)	
* human metapneumovirus*	7 (10.0%)
* respiratory syncytial virus (RSV)*	2 (2.9%)
* rhino-/enterovirus*	5 (7.1%)
* influenza A*	8 (11.4%)
* influenza B*	4 (5.7%)
* Varicella-zoster virus (VZV)*	7 (10.0%)
* Coronavirus*	1 (1.4%)
* Herpes simplex virus 1 (HSV 1)*	1 (1.4%)
* Human Parainfluenza virus*	1 (1.4%)
bacterial co-infections, n (%)	7 (10.0%)
* Streptococcus pneumoniae*	2 (2.9%)
* Staphylococcus aureus/hominis*	1 (1.4%)
* Streptococcus agalactiae*	1 (1.4%)
* Moraxella catarrhalis*	2 (2.9%)
* Haemophilus influenzae*	1 (1.4%)
multiple co-infections, n (%)	4 (5.7%)
* human Metapneumovirus* + *Rhino/Enterovirus*	1 (1.4%)
* Influenza A* + *Staphylococcus* *aureus/hominis*	1 (1.4%)
* Rhino-/Enterovirus* + *VZV*	1 (1.4%)
* Influenza A* + *Haemophilus* *influenzae*	1 (1.4%)

Values are n (%) unless stated otherwise

Testing strategies (and timing) were not standardized across centres; interpret between group comparisons with caution

## Discussion

This nationwide, multicentre cohort describes children and adolescents admitted to Austrian PICUs with iGAS during 2022–2024. We observed a marked winter-spring seasonality, frequent respiratory presentations (particularly pleural empyema and pneumonia), and substantial need for advanced organ support. Overall mortality was 14.3%.

We observed a predominance of younger children, with a median age of 4.5 years. This aligns with reports from other European countries, where the 2022 iGAS increase mainly affected children < 5–10 years [20, 21]. Similar findings were reported in Australia (median age 4.5 years; > 60% < 5 years) [27] and Denmark (> 50% aged 0–4 years) [16]. In the EUCLIDS cohort, younger age (median 40 months) was associated with PICU admission but not disability [11]. Only 3 of 70 patients (4.3%) in our cohort were < 1 year, limiting conclusions for this subgroup.

Reports from Europe and the UK documented a marked rise in paediatric iGAS beginning in late 2022, with increased burden in children under 10 years, framing the period of this study [15, 20, 21, 23–25, 28, 29]. The surge in 2022–23 is considered multifactorial, occurring after historically low GAS circulation during pandemic restrictions [16, 22, 30, 31]. Unusually intense and off-season outbreaks of respiratory viruses were observed after restrictions were lifted [32, 33]. The broader rise in viral infections across Europe and the USA likely contributed, as preceding viral illnesses such as respiratory infections and varicella infections increase the susceptibility to iGAS [13, 16, 18, 21, 34–37]. In England, increased iGAS infections with respiratory viral co-infection among children under 15 years was reported in October–November 2022 [21].

In our cohort, 39/70 patients (55.7%) had a documented co-infection, although centre-level differences in testing limit causal inference. These findings support vigilant testing for co-pathogens in children with suspected iGAS and reinforce the potential indirect value of maintaining high varicella immunisation coverage. Compared with an Australian cohort [27], in which rhino-/enterovirus, human metapneumovirus and adenovirus predominated, our series more often identified human metapneumovirus, influenza A and rhino-/enterovirus among co-infected patients. Importantly, Abo et al. reported that respiratory viral positivity was associated with more severe disease [27].

Respiratory symptoms dominated initial presentation in our cohort: pleural empyema (51.4%), pneumonia (45.7%), and sepsis (44.3%), with septic shock being present in 25.7%. This pattern mirrors recently published series describing increased lower-respiratory involvement, particularly pleural disease, during the surge, and contrasts with some pre-pandemic cohorts in which bacteraemia without an identifiable focus and cellulitis were more common [2, 7, 38–42]. Differences likely reflect temporal shifts in co-circulating pathogens, referral patterns to the PICU, and heightened diagnostic sampling of pleural fluid in critically ill children.

The need for organ support was high in this study cohort. Mechanical ventilation was required in 72.9% (44.3% invasive, 28.6% non-invasive), vasoactive therapy in 50%, and CPR in 14.3%. The use of extracorporeal therapies was also substantial: ECMO in 7.1% (all VA-ECMO) and CRRT in 7.1%, which is higher than in some pre-pandemic European cohorts but comparable to contemporary Australian data [11, 27]. Broman et al. note that, according to guidelines from The American College of Critical Care Medicine and the Society of Critical Care Medicine, ECMO is recommend for refractory paediatric septic shock in experienced centres [43, 44]; our findings reinforce the need for early recognition of cardiovascular failure and development of regional escalation pathways.

Microbiologically, most confirmations came from sterile sites, particularly pleural fluid and blood. Multi-site positivity was common (40.0%). These observations align with UK surveillance data noting increased detection from pleural fluid and other lower-respiratory specimens during the surge [11, 21]. Because specimen collection was not standardised, these comparisons should be interpreted cautiously.

In the EUCLIDS GAS cohort, mortality among PICU-admitted children was reported 6/148 (4%), whereas mortality in our PICU-only cohort was 10/70 (14.3%) [11]. This comparison is limited by differences in study design, inclusion criteria, case mix and study period.

Notably, higher early mortality (often cited as 8–23%) pertains to severe presentations, particularly streptococcal toxic shock syndrome, and differs from overall paediatric case-fatality estimates in high-income settings [2, 5–8, 38–42]. Clinically, our data highlight three priorities: prompt recognition of iGAS with respiratory focus, early hemodynamic support with readiness to escalate to ECMO in refractory shock, and systematic assessment for respiratory viral and varicella co-infection.

A topic not addressed in this study is strain-level virulence. The most important virulence factor of GAS is the M protein, encoded by the *emm* gene, which also constitutes its major surface protein [45]. The M protein plays a central role in host colonization, resistance to phagocytosis, and the ability to invade normally sterile areas [46–48]. The extensive genetic variability of the M protein, with more than 200 *emm*-types identified, is thought to contribute significantly to the wide range of clinical manifestations associated with GAS infections [11, 30, 31, 47, 49–51]. Future studies should incorporate emm-typing and link type distribution to clinical severity in invasive paediatric cases.

### Strengths and limitations

Strengths of this study include nationwide coverage across all 13 Austrian PICUs during the period of highest paediatric iGAS activity, granular clinical detail, and consistent stratification by a priori-defined outcome groups.

Limitations include the retrospective design, potential selection bias towards more severe respiratory phenotypes admitted to PICU, non-standardised testing across centres and over time, precluding robust multivariable analysis. The cohort was restricted to PICU admissions; therefore, children managed outside the PICU or those who died before PICU admission were not captured, potentially underestimating the number of the most severe iGAS cases.

Validated severity-of-illness scores were not collected retrospectively, as they are not routinely standardized across Austrian PICUs; therefore, disease severity was described using clinical variables and organ-support requirements. Diagnoses of initial leading clinical presentations, including sepsis, septic shock and pARDS, were retrospectively collected across multiple PICUs without central verification of consistent diagnostic criteria. Another limitation is the retrospective reporting of vasoactive support without a standardized operational definition regarding type, dose or duration of vasoactive or inotropic medication, potentially reducing consistency across centres***.***

In addition, only positive microbiological test results were recorded, while negative results and total tests performed were unavailable, precluding denominators and evaluation of diagnostic test performance. Vaccination status, including for varicella and influenza, was not available and therefore could not be incorporated into the analysis of vaccine-preventable co-infections. Furthermore, emm-typing, antimicrobial and IVIG data, and long-term outcomes were unavailable, and population incidence could not be estimated.

## Conclusion

During 2022–2024, paediatric iGAS requiring PICU admission in Austria was characterised by predominantly respiratory presentations, frequent organ-support requirements, and notable mortality. These findings mirror international trends and emphasise the need for coordinated prevention, early recognition, and timely access to advanced critical-care support, including extracorporeal rescue in experienced centres when clinically indicated [43, 44].

## Data Availability

All data supporting the findings of this study are available within the paper.

## Abbreviations

CPR: Cardiopulmonary resuscitation
CRRT: Continuous renal replacement therapy
DIC: Disseminated intravascular coagulation
EUCLIDS: European Union Childhood Life-threatening Infectious Disease Study
ECMO: Extracorporeal membrane oxygenation
HPSC: Health Protection Surveillance Centre
iGAS: Invasive *Group A Streptococci*
IQR: Interquartile range
pARDS: Paediatric acute respiratory distress syndrome
PCR: Polymerase chain reaction
PICU: Paediatric Intensive Care Unit
PICUs: Paediatric Intensive Care Units
STSS: Streptococcal toxic shock syndrome
UK: United Kingdom
VA-ECMO: Veno-arterial extracorporeal membrane oxygenation
VV-ECMO: Veno-venous extracorporeal membrane oxygenation
WHO: World Health Organization
